# Mindfulness and self-regulation intervention for improved self-neglect and self-regulation in diabetic older adults

**DOI:** 10.1038/s41598-024-64314-y

**Published:** 2024-06-15

**Authors:** Mohadeseh Motamed-Jahromi, Mohammad Hossein Kaveh, Elsa Vitale

**Affiliations:** 1 Department of Public Health, School of Health, Fasa University of Medical Sciences, Fasa, Iran; 2 Research Center for Health Sciences, Department of Health Promotion, School of Health, Institute of Health, Shiraz University of Medical Sciences, Razi Boulevard, P.O. Box: 7153675541, Shiraz, Iran; 3 Mental Department, Asl Bari, Bari, Italy

**Keywords:** Mindfulness, Self-regulation, Self-neglect, Diabetes mellitus, Aged, Psychology, Diseases, Health care

## Abstract

The current study aimed to assess the impact of combined interventions including mindfulness and self-regulation on self-neglect and self-regulation among Iranian older adults with type 2 diabetes. This was a three-arm cluster randomized controlled trial study conducted among 135 older diabetic patients in Shiraz, Iran. Three urban healthcare centers (clusters) were randomly assigned to three study groups. The intervention groups received either a Self-Regulation-based Intervention Program (SRIP) or a Combined Mindfulness and Self-Regulation Intervention Program (CMSRIP), while the control group received routine care and COVID-19 prevention training. These training programs, which consisted of text and video-based content, were conducted over 24 weeks using WhatsApp as a mobile-based communication platform. Outcomes were measured using the Elder Self-Neglect Scale and Short-Form Self-Regulation Questionnaire at baseline, week 4, and week 16 post-intervention, with data analysis conducted using SPSS _20_ software. The CMSRIP led to significantly greater improvement in the score of self-regulation (χ^2^ = 73.23, *P*-Value =  < .001) and a reduction in the score of self-neglect (χ^2^ = 62.97, *P*-Value =  < .001) at both 4 weeks and 16 weeks after education compared to SRIP. In the control group, there was also a slight improvement. Improvement of self-regulation and reduction of self-neglect in all three groups were less in week 16 than in week 4. Nevertheless, the changes in the intervention groups were significantly better than the control group. This study confirmed a combination of mindfulness-based intervention and self-regulation intervention can effectively improve self-neglect and self-regulation behavior in older patients with type 2 diabetes.

Trial registration: This trial (ISRCTN77260130) was retrospectively registered on 28/09/2021.

## Introduction

Population aging is a significant global trend, particularly pronounced in Asian countries due to their large populations^1^. In 2013, the proportion of people aged 65 and over in Asia was 7.11%, projected to increase to 14% by 2036^2^. Iran has also experienced rapid growth in its older population, with the percentage rising from 8.2% in 2011 to 9.3% in 2016^3^. This demographic shift poses challenges for successful aging and managing prevalent chronic diseases among the older population^4^.

Diabetes is indeed a common chronic disease among older people, leading to challenges in self-care due to factors like decreased physical and cognitive function associated with aging^5,6^. The physiological and pathological changes linked to aging further compound the difficulties in self-care and disease management for older people with diabetes^7^. In addition, one of the important obstacles to increasing the use of self-care methods in diabetic older adults is the phenomenon of self-neglect^8^.

Self-neglect among older adults is a serious issue involving incapacity or refusal to perform essential self-care tasks, reducing personal resources and motivation for self-care^9–11^. This issue can have severe consequences, threatening health and safety^12^. Various predisposing factors contribute to self-neglect, including mental illness, pre-illness personality traits, low physical activity levels, reduced social interaction, alcohol abuse, and low socioeconomic status^12^. Research has shown that executive dysfunction in the aging process, which involves the inability to complete complex tasks and manage daily activities, is strongly associated with self-neglect^13,14^.

The decline in self-care among diabetic patients is also linked to a lack of self-regulatory resources, underscoring the importance of establishing self-care practices before dysfunctional behaviors become habitual^15^. Self-regulation is depicted as a cyclical process that encompasses task analysis, goal setting, strategy development, strategy application, and action monitoring in a metacognitive manner^16,17^. This process necessitates voluntary control of emotions, attention, and behavior to attain personal goals and standards^18^.

It appears that interventions targeting self-neglect and self-regulation in older adults with diabetes are indeed essential to improving self-care and diabetes management. Among the various interventions, self-regulation interventions, as a behavioral approach, have shown positive effects on self-care behaviors in older people with diabetes^19^. Studies have shown that self-regulation, which includes aspects like self-efficacy, knowledge, social support, and outcome expectations, plays a crucial role in diabetes self-management among the older population^20,21^. In addition, enhanced self-regulation can contribute to developing healthier coping mechanisms, improved decision-making abilities, and better emotional regulation, all of which are vital for addressing and preventing self-neglect^22^.

Self-regulation is indeed a problem-solving process where the mind plays a central role^23^. To function effectively, the mind needs to be organized and free from disruptions like preoccupied-mind syndrome or cognitive distortions^24^. Mindfulness practices, commonly used in cognitive-behavioral therapy, can help organize and enhance thinking processes by freeing the mind from unhelpful thoughts, ultimately enhancing the capacity for self-regulation^25^. Mindfulness training has been linked to improvements in emotion regulation, executive functions, and neurocognitive control processes, all of which contribute to enhanced self-regulation abilities across various domains of life^26^. This enables individuals to manage emotions, make intentional decisions, and respond to challenges with patience and mindfulness^27^.

Additionally, Mindfulness interventions are effective in enhancing self-care and psychological well-being in individuals, particularly in the context of managing chronic conditions like diabetes^28^. Mindfulness, through enhancing self-awareness and emotional regulation, fosters a present-focused approach by observing internal experiences without judgment^29^. This practice allows individuals to become more attuned to their needs, promoting a balanced self-care approach and addressing self-neglect in chronic conditions^27^.

Based on the literature review, past experimental studies have only focused on evaluating the effect of self-regulation or mindfulness in diabetic older adults. Specifically, a study by Jiang et al. focused on older people with diabetes and indicated positive outcomes related to mindfulness practices in managing diabetes and improving overall well-being among diabetic older adults^23^. Another study evaluated the effectiveness of a mindfulness program for older adults with type 2 diabetes living in long-term care facilities, showing positive effects of the mindfulness program on emotional well-being and self-regulation in this group^24^ Additionally, the study by Li et al. in 2023 found that self-regulation interventions for diabetes management in older people and found that interventions led to increased knowledge, attitude, and self-management behavior scores over time in the experimental group^25^. Another study demonstrated that tailored self-management training programs designed for diabetic older adults can lead to significant improvements in self-management behaviors and glycemic control^26^.

It seems that the combination of mindfulness exercises and self-regulation-enhancing skills may lead to higher outcomes in terms of reducing self-neglect and increasing self-regulation. Based on the evidence, combined intervention programs that integrate various components reduce comorbidities and improve psychological and physical capacities in older adults^30^. Therefore, the integration of mindfulness and self-regulation programs offers a synergistic approach that can lead to enhanced outcomes for older diabetic patients^31^. By combining these approaches, a more comprehensive intervention is achieved, addressing cognitive, emotional, and behavioral aspects to maximize the benefits of each approach^32^.

The study hypothesizes that the integration of mindfulness and self-regulation interventions in older diabetic patients will result in a significant reduction of self-neglecting behaviors and a notable improvement in self-regulation capabilities. This combined intervention approach is expected to positively impact cognitive processes, enhance executive brain function, and empower cognitive and behavioral aspects simultaneously, leading to improved self-care outcomes and overall well-being in this population. The innovation of the study lies in integrating mindfulness and self-regulation interventions to address self-neglect and enhance self-regulation in Iranian diabetic old patients. The study aims to provide a holistic method for improving cognitive and behavioral empowerment simultaneously in diabetic older adults by combining mindfulness and self-regulation interventions. This novel approach not only fills a gap in the existing literature but also offers a comprehensive strategy to prevent self-neglect and promote sustainable self-management in a specific population, highlighting the study's significant contribution to advancing psychiatric care for older diabetic patients.

## Methods

### Study design

A 24-week, double-blinded, three-arm cluster randomized control trial with 2 intervention arms and one control arm was conducted at three urban health centers in Shiraz, Iran. In this study, participants and analysts were blinded. Participants were sampled from three separate health centers in different regions and did not know which group they were in. The main analyst was a person outside the study team and only knew the studied groups as groups one, two, and three. This study was conducted under the conditions of the COVID-19 pandemic. Therefore, the WhatsApp platform was utilized for educational interventions for participants. Additionally, phone calls were employed for questionnaire completion, and WhatsApp video calls were used to assess which older adults met the inclusion criteria for the study.

### Participants and Sampling process

To protect against contamination by participants from the same community, one urban healthcare center in Shiraz was taken as a cluster in the randomization process. Typically, Shiraz has 16 urban healthcare centers, with 8 of them specifically providing care for diabetics. In the study, three out of the eight urban healthcare centers in Shiraz were chosen by simple random sampling and allocated to three arms of the study. Center 1 hosted the self-regulation-based intervention program (SRIP) group, Center 3 hosted the combined mindfulness and self-regulation intervention program (CMSRIP) group, and Center 2 hosted a group receiving routine care with COVID-19 prevention training as the control group. Eligible older patients with diabetes were then selected from each center (n = 45) using a simple random sampling method. In this study, Center 3 had 173 eligible people, Center 1 had 115 eligible people, and Center 2 had 84 eligible people. From each center, 45 people were randomly included in the study.

It was estimated that 45 participants would be placed in each group using Power Analysis and Sample Size (PASS) software (version 11.0.7, PASS, NCSS, LLC) with a 0.32 effect size at 0.05 alpha level, the mindfulness mean score ± SD at 118.3 ± 7.40, and the possibility of a 10% sample loss^33^. The parameters used in the calculation were derived from Zhou et al.^34^. The inclusion criteria for the study encompassed individuals aged between 60 and 80 years old to establish a more uniform sample with similar needs. Additionally, participants needed to have type 2 diabetes with a duration of at least 6 months to ensure they had experience managing the condition. Other criteria included at least an elementary level of literacy, normal cognitive status, basic activities of daily living (personal hygiene, dressing, toileting, transferring, and eating), access to a smartphone and the internet, and self-rated ability to use WhatsApp for sending and receiving plain text and video messages. The exclusion criteria for the study involved persistent serious psychological issues, individuals not responding to phone calls and messages after three attempts, and reluctance to engage in the intervention. Cognitive ability was assessed using the Mini-Mental State Examination (MMSE) as an inclusion criterion. The MMSE was conducted via WhatsApp video call, taking less than ten minutes per person. A score between 24 and 30 on the MMSE indicated normal cognitive health, and participants with normal cognitive status were included in the study. The mean MMSE scores for both the intervention and control groups are detailed in Table 2^35^. To determine eligible individuals for the study, files in the healthcare centers, phone calls, and video calls were utilized.

### Structural intervention

The 6-month training period from December 2020 to June 2021 involved participants from all three groups receiving training through WhatsApp. Initially, messages were sent daily to participants for the first two months and then reduced to only Mondays for the following four months. This training approach aimed to engage participants consistently over time and deliver training content effectively. For each part of the training, forms were prepared based on the training content in mindfulness training and given the needs and problems of patients in self-care training based on self-regulation theory. Examining the forms and participants' feedback that week indicated their progress in learning. An example of forms and feedback are in supplementary files (Appendix 1). Participants were assessed at the baseline, 4 weeks, and 16 weeks after daily intervention. SRIP and CMSRIP groups consisted of 8 and 16 modules in the first two months, respectively. The modules included text, images, songs, audio, and video, emoticons, links, and interactive assignments related to mindfulness or self-regulation. The purpose of the messages was to encourage, remind, and give feedback.

In the SRIP group, the educational content of 8 weeks was adjusted according to the self-care needs of diabetic patients by observing the structures of self-regulation theory. These constructs include self-awareness, goal setting, action planning, self-monitoring, and feedback^36^. The training was provided to the older people in the form of texts, audio and videos, and educational links. The CMSRIP group implemented two interventions over eight weeks, consisting of 16 modules. These interventions included mindfulness training based on the work of Segal et al.^37^ and self-regulatory training similar to the SRIP group. The focus was on cognitive reconstruction and enhancing performance through these structured interventions. The initial focus in the first 4 weeks of the program was on mindful self-care techniques to improve cognitive processes related to decision-making and self-care behavior change. Participants were offered appropriate educational videos, nature sounds, and Persian songs weekly, following the stages outlined in Segal et al.'s approach. An overview of all the modules can be seen in Table 1. All participants in the groups provided weekly feedback on a specific day of the week and received instructor feedback. Then, tailored interventions in both groups were continued based on the groups' goal every Monday for 4 months. In the control group, participants received COVID-19 prevention training as well as routine training at the same time as the previous two groups. The control group, the CMSRIP group, and the SRIP group received 293, 287, and 240 messages, respectively, over six months. Information through telephone interviews was collected at baseline, week 4, and week 16 after daily education.

**Table 1:** Details of interventions in three groups of study.

Mindfulness + self-regulation program	Mindful program based on the book by Segal et al.		• Four-square breathing, raisin exercise, body scan meditation
			• Mindful walking meditation, 10-min sitting meditation
			• Lake meditation, mountain meditation
			• Seeing" and "hearing" exercise, mindful walking, sitting meditation
			• Mindfulness STOP skill
			• 30-min sitting meditation with awareness of breath, body, sounds and thoughts/feelings • Audio meditation, silent meditation to the sound the bell
			• The self-compassion pause, 5 steps to boost self-confidence
	Self-regulation program only	Based on self-regulation theory	• Need assessment
			• Diet training
			• Diabetes medication management
			• Teaching stress reduction and smoking cessation skills
			• Physical activity training
			• Foot care
			• Supplements and herbal and traditional medicines • Training on using a glucometer and insulin injection technique
		Control group	• Routine care
			• COVID-19 prevention program

### Measures

Study data were collected using three questionnaires. These three included the demographic information form, the elder self-neglect scale (ESNS), and the short-form self-regulation questionnaire (SSRQ).

### Demographic information form

Data on demographic characteristics of the participants were collected by using an index-type of items including short-answered and force-choice questions. Variables to be measured were gender, age, marital status, education level, occupational status, living arrangement, other diseases, duration of diabetes, and cognitive ability.

### Elder self-neglect scale (ESNS)

Motamed-Jahromi et al. conducted a study where they developed and validated the Elder Self-Neglect Scale (ESNS) for Iranian older adults^38^. In developing process of this scale to measure elder self-neglect, related literature, and similar tools were reviewed by the research team and a new scale was developed tailored to the study population. Items were rated on a 5-point Likert rating scale, ranging from 1 (never) to 5 (always) based on the frequency of the behavior. After that, we assessed content, face and construct validity, and internal consistency of scale with the participation of 700 community-dwelling old population. Exploratory and confirmatory factor analysis was performed in the construct validity process. After exploratory factor analysis, our scale consisted of 26 items and 6 dimensions (physical environment, physical health, mental health, financial status, social network, and self-determinant). The total score was a sum of the mean of the scores between 26 and 130 and a lower score indicated more severe elder self-neglect. After confirmatory factor analysis, the scale’s fit indices were optimal (RMSEA = 0.04, GFI = 0.90, CFI = 0.95, CMIN/DF = 1.48). Internal consistency was assessed and Cronbach's alpha coefficient was obtained at 0.85. The test–retest reliability of ESNS administered to a subsample of 30 older adults with a one-month interval was 0.73, indicating good temporal stability.

### The short form self-regulation questionnaire (SSRQ)

The Short Form Self-Regulation Questionnaire SSRQ developed by Carey et al., 2004 was used to assess self-regulation behavior in diabetic older adults. It included 31 items that were scored on a 5-point Likert scale, ranging from 1 (strongly disagree) to 5 (strongly agree), and were summed items to create a total score^39^. Carey et al. in 2004 reported that the SSRQ measured only one dimension^40^. Motamed-Jahromi et al. confirmed the validity and reliability of the Persian version of the Short Form Self-Regulation Questionnaire (SSRQ) for use in Iranian community-dwelling older adults. This new version was shorter and included 20 items with 4 dimensions: self-awareness (6 items), goal-setting (2 items), action planning (6 items), and self-monitoring (6 items). The scale's fit indices were optimal (RMSEA = 0.059, GFI = 0.89, CFI = 0.92, and CMIN/DF = 1.87) and the internal reliability of SSRQ was also good (Cronbach α = 0.78). The test–retest reliability of the SSRQ was measured by the correlation coefficient between the two completion times and was 0.68 for a subsample of 30 participants. The total score was the sum of the scores of the items (between 20 and 100). The higher the scores indicated a better level of self-regulation^41^.

### Data analysis

Statistical analyses were performed using Statistical Package for Social Sciences (SPSS) software Version 20.0 (IBM SPSS, SPSS Inc., Chicago, IL, USA). A descriptive statistical analysis was applied to determine the demographic profile of the participants. ANOVA test was applied to ‘duration of diabetic disease’ and ‘cognitive ability’ (Mean ± SD), as well as chi-square tests, were used to assess the rest demographic variables (number (percent)). The Kolmogorov–Smirnov demonstrated the data has a normal distribution. General linear model (GLM) repeated measures were used to compare the mean change in variables adjusted to the baseline score at a confidence interval of 95%. Eta Squared was used to calculate the effect size. Post hoc paired comparisons were conducted using the Bonferroni test to determine the changes in mean scores between different groups at different time points. Bar charts were drawn using Microsoft Excel 16 to illustrate the differences between the mean of various dimensions of two questionnaires at specific time points. Significant at the level of *P* < 0 0.05 was considered.

### Ethical consideration

This study was approved by the Ethics Committee of the Shiraz University of Medical Science (Ref. no: IR.SUMS.REC.1398.1365), which is available from: https://ethics.research.ac.ir/. The trial registration number of this study is ISRCTN77260130, which is available from: https://www.isrctn.com/ISRCTN77260130. The study adheres to the ethical principles outlined in the Declaration of Helsinki by the World Medical Association. Furthermore, all methods employed in the study were conducted by the relevant guidelines and regulations, ensuring ethical standards were upheld throughout the research process. All participants were informed that participation in the study was voluntary. Then, all participants gave informed consent. They were also assured that the data collected would remain confidential.

## Results

### Participants’ characteristics

**Figure1:**
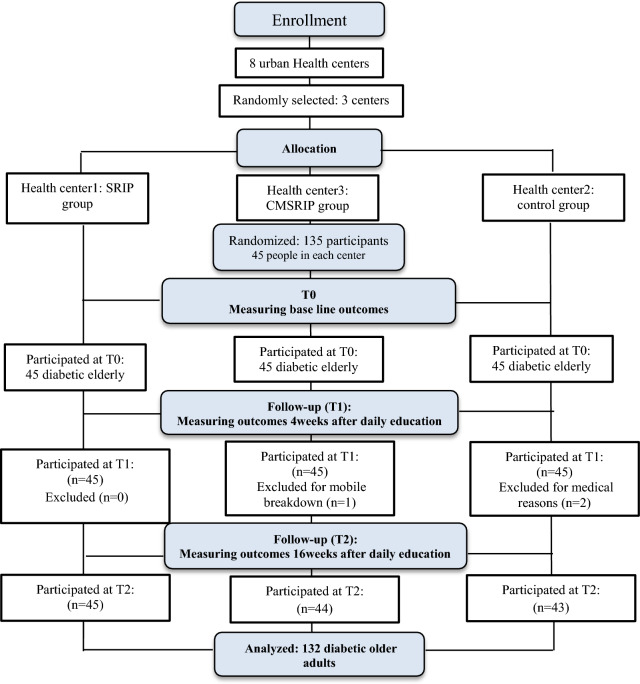
Consort diagram of study participants: T0, baseline; T1, week-4; T2, week-16.

In total, 132 out of 135 diabetic old people completed the study. The response rate in the study was 97%. Within the control group, two individuals were unable to finish the study due to medical reasons. Furthermore, one participant from the CMSRIP group was excluded from the study for breaking a cell phone. Figure 1 presents a CONSORT diagram for the study.

In terms of participant characteristics, there was no statistically significant difference between the intervention and control groups. The mean age of study participants was 66.42 ± 5.35 years and 74 out of 132 (55.9%) old people were females (Table 2).

**Table 2:** Demographic profile of participants in the study groups.

	SRIP group (n = 45)	CMSRIP group (n = 44)	Control (n = 43)	*P* value
	N (%)	N (%)	N (%)	
*Sex*				
Male	15 (33.3%)	20 (45.5%)	23 (53.5%)	0.161
Female	30 (66.7%)	24 (54.5%)	20 (46.5%)	
*Age*				
60–70	33 (73.3%)	33 (75.0%)	32 (74.4%)	0.970
70–80	11 (24.4%)	8 (17.2%)	9 (20.9%)	
80^+^	1 (2.2%)	3 (6.8%)	2(4.7)	
*Marital status*				
Single	1 (2.2%)	2 (4.5%)	2 (4.7%)	0.264
Married	38 (84.4%)	39 (88.6%)	39 (90.7%)	
Divorced/separated/widow	6 (13.3%)	3 (6.8%)	2 (4.7%)	
*Education*				
High school grade(diploma) or less	38 (84.5%)	29 (65.9%)	31 (72.1%)	0.052
Academic education	7 (16.5%)	15 (34.1%)	12 (27.9%)	
*Occupational status*				
Self-employed	12 (26.7%)	7 (15.9%)	12 (27.9%)	0.157
Civil servant	3 (6.7%)	1 (2.3%)	7 (16.3%)	
Retired	9 (20.0%)	18(40.9%)	11 (25.6%)	
Housewife	21 (46.7%)	18 (40.9%)	13 (30.2%)	
*Other disease*				
Musculoskeletal diseases	13 (28.9%)	15 (34.1%)	11 (25.6%)	0.345
Cardiovascular disease	17 (37.8%)	7 (15.9%)	14 (32.6%)	
Hypertension	9 (20.0%)	9 (20.5%)	10 (23.32%)	
Depression	1 (2.2%)	1 (2.3%)	1 (2.3%)	
Other	5 (11.1%)	12 (27.3%)	7(16.3)	
*Living arrangement*				
With family or relatives	36 (80.0%)	35 (77.3%)	35 (81.4%)	0.891
Living alone	9 (20.0%)	10 (22.7%)	8(18.6%)	
	Mean ± SD	Mean ± SD	Mean ± SD	
Duration of diabetic disease	6.02 ± 3.78	5.86 ± 3.20	6.40 ± 3.48	0.768
Cognitive ability (using MMSE)	25.9 ± 4.7	26.7 ± 3.4	27.5 ± 3.2	0.492
Significant at the level of *P* < 0 .05				

### Outcomes

Table 3 compares the scores of ESNS and SSRQ between the intervention and control groups at baseline, one month after the intervention, and four months after the intervention. The results of the generalized linear model (GLM) test indicated that the combined mindfulness and self-regulation intervention program (CMSRIP) had a significant effect on the self-regulation variable over time, with a higher effect coefficient compared to other groups (*P* < 0.001, effect size = 0.808). Similarly, the self-regulation intervention program (SRIP) also showed a significant effect on the self-regulation variable (*P* < 0.001, effect size = 0.537). Additionally, the COVID-19 prevention intervention significantly impacted the self-regulation of older adults with diabetes in the control group (*P* < 0.001, effect size = 0.627). Comparison between groups in the mean scores of the self-regulation variable at baseline did not show a statistically significant difference in the intervention and control groups, and all groups had almost the same mean score before the intervention (*P*-value = 0.612).

**Table 3:** Comparison of the SSRQ and ESNS scores of intervention and control groups at different time points.

Scale	Groups	Mean (SD)			*P* value*	Effect size (eta squared)
		Baseline	1 month after the intervention	4 months after the intervention		
Short-form self-regulation questionnaire (SSRQ)	CMSRIP	55.36(9.76)	73.89(4.18)	68.84(3.39)	< .001	0.808
	SRIP	57.76(13.61)	70.40(6.75)	66.18(4.52)	< .001	0.537
	Control	57.04(6.35)	65.93(4.40)	63.73(7.28)	< .001	0.627
*P*-value**		0.612	< .001	< .001		
Elder Self-neglect scale (ESNS)	CMSRIP	102.67(6.72)	75.73(13.96)	80.13(10.70)	< .001	0.836
	SRIP	100(11.24)	81.42(6.28)	83.07(10.4)	< .001	0.630
	Control	101.11(8.69)	89.13(7.25)	95.13(10.98)	< .001	0.579
*P*-value**		0.763	0.002	< .001		

CMSRIP: combined mindfulness and self-regulation intervention program group, SRIP: self-regulation intervention program group. Significant at the level of P < 0 .05. *within-group, **between-group.

The combined mindfulness and self-regulation intervention program (CMSRIP) demonstrated a significant effect on the self-neglect variable over time, with a higher effect coefficient compared to other groups (*P* < 0.001, effect size = 0.836). Similarly, the self-regulation intervention program also had a significant effect on the self-neglect variable (*P* < 0.001, effect size = 0.630). Furthermore, the COVID-19 prevention intervention significantly impacted the self-neglect variable among older adults with diabetes in the control group (*P* < 0.001, effect size = 0.579). The comparison between groups in the mean scores of the self-neglect variable at baseline did not reveal a statistically significant difference between the intervention and control groups, and all groups exhibited nearly identical mean scores before the intervention (*P*-value = 0.763).

**Figure 2:**
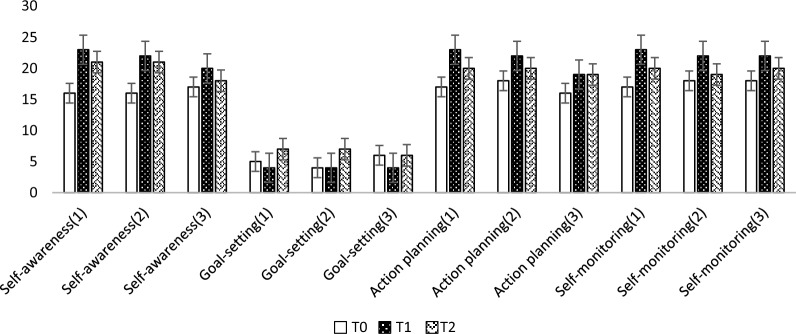
Mean scores of SSRQ dimensions at baseline (T0), 1 month after intervention (T1), and 4 months after intervention (T2) in three groups. Groups: 1 = CMSRIP, 2 = SRIP, and 3 = control.

**Figure 3:**
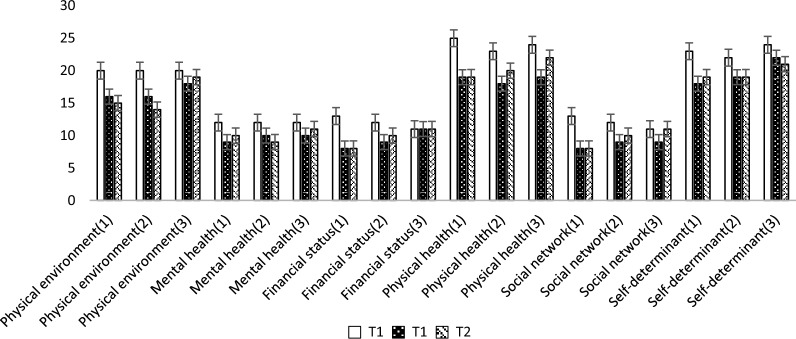
Mean scores of ESNS dimensions at baseline (T0), 1 month after intervention (T1), and 4 months after intervention (T2) in three groups. Groups: 1 = CMSRIP, 2 = SRIP, and 3 = control.

Figures 2 and 3 illustrate the mean scores of the various dimensions of ESNS and SSRQ in three study groups at baseline, week 4, and week 16 following daily interventions. Figure 2 indicates an increasing trend in mean scores for self-regulatory dimensions after the interventions across all three groups, with a slight decrease observed by week 16. Notably, the goal-setting dimension displayed an exception, with mean scores lower than the baseline in all three groups at week 4 post-intervention. Figure 3 demonstrates a decreasing trend in mean scores across different ESNS dimensions at week 4 and week 16 compared to the baseline, with a slight increase in scores noted by week 16.

Table 4 presents the outcomes of Bonferroni's post hoc test. The results indicate significant changes from baseline to week 4 and baseline to week 16 in mean scores of ESNS and SSRQ in all three groups. Moreover, significant alterations in mean scores of SSRQ were observed from week 4 to week 16 in CMSIRP and SIRP groups. Additionally, changes in mean scores of ESNS from week 4 to week 16 were found to be significant only in the control group.

**Table 4:** Pairwise comparisons of mean scores of SSRQ and ESNS using Bonferroni test.

	Group	Change from baseline to week-4	Change from baseline to week-16	Change from week-4 to week-16
ESNS	SRIP	18.57 ± 2.19	16.93 ± 2.45	− 1.64 ± 2.09
	p-value	< .001	< .001	1
	CM SRIP	26.93 ± 2.33	22.53 ± 1.86	− 4.40 ± 2.44
	*p*-value	< .001	< .001	0.236
	Control	11.97 ± 1.59	5.97 ± 2.09	− 6.00 ± 1.71
	*p*-value	< .001	0.019	0.003
SSRQ	SRIP	− 12.64 ± 2.10	− 8.42 ± 2.06	4. 22 ± 0.824
	*p*-value	< .001	0.001	< .001
	CM SRIP	− 18.53 ± 1.49	− 13.48 ± 1.41	5.04 ± 0.547
	*p*-value	< .001	< .001	< .001
	Control	− 8.88 ± 1.03	− 6.68 ± 1.45	2.20 ± 1.21
	*p*-value	< .001	< .001	0.228

Significant at the level of *P* < 0.05.

## Discussion

Identifying strategies to establish effective and continuous self-care and improve self-neglect is a significant challenge in managing diabetes. These strategies empower individuals to manage their condition, prevent complications, and enhance their quality of life. The study aimed to compare the impact of a self-regulation intervention alone with combined interventions of mindfulness and self-regulation on self-neglect and self-regulation in Iranian diabetic older adults.

One of the key findings of the trial was that the combined interventions of mindfulness and self-regulation led to a more significant improvement in diabetic older patients’ self-regulation compared to the self-regulation intervention alone. This enhancement can be attributed to the synergistic effects of mindfulness and self-regulation on improving self-care behaviors among older adults with type 2 diabetes^31^. Leyland et al. highlighted that mindfulness practices, such as attentional changes, present moment awareness, and regulation of negative emotions, can enhance executive function and influence the utilization of self-regulation strategies, leading to improved efficiency in managing diabetes among older adults^26,42^. Ultimately, this comprehensive approach focusing on cognitive and behavioral empowerment is a tailored and effective strategy for managing diabetes in older people.

In this study, the effect of integrated interventions of mindfulness and self-regulation in reducing self-neglect among older people, especially people with diabetes, was a valuable finding. Older adults with diabetes encounter difficulties in self-care due to cognitive and physical decline, highlighting the importance of interventions to prevent complications^43^. Mindfulness interventions target negative thoughts and behaviors, while self-regulatory strategies gradually empower older people^44,45^. Studies have demonstrated that these combined interventions can improve self-care, leading to a reduction in self-neglect among older adults with diabetes^46,47^. The researchers also believed that mindfulness interventions can lead to self-compassion and psychological health and confirmed mindfulness is an important mediator in improving self-care and self-awareness^48,49^.

The effect of combined mindfulness and self-regulation interventions on the self-regulation of older people with diabetes was an apparent result. These interventions improved self-awareness, action planning, and self-monitoring, especially in the CMSRIP group. For older people with diabetes, self-regulation enables them to make informed health decisions, enhancing disease management, and quality of life, and reducing complications associated with diabetes^50^. The positive influence of mindfulness on psychological outcomes and glycemic control underscores its value as a beneficial adjunct therapy for older people with diabetes, emphasizing the importance of integrating mindfulness practices into diabetes self-regulation programs^51,52^.

An unexpected finding was a minor change in the goal-setting dimension in the self-regulation questionnaire in the three groups 4 weeks after the daily interventions. This lack of change can be attributed to short-term factors such as anxiety from receiving a lot of information and low self-management skills, which hindered the decision-making and goal-setting process initially. However, over the long term, specifically 16 weeks after the daily intervention, goal-setting improved due to increased experience, reduced anxiety, and enhanced ability. Studies have shown that one crucial strategy in managing diabetes in older people involves emphasizing the significance of setting clear goals by simplifying care plans and leveraging the education and experience of successful disease management^53,54^.

The findings also indicated that the most significant changes in scores across all three groups occurred in the 4 weeks following daily interventions, highlighting the short-term impact of these interventions. Furthermore, the changes in scores from the two questionnaires persisted into the 16 weeks following daily interventions at a slower rate, indicating long-term effects. As educational sessions were only provided on Mondays over the next 16 weeks, these sustained changes are likely attributed to the continuous educational support and the chance for older adults to enhance their skills and experiences. Therefore, a well-structured continuing education program can sustain long-term changes, leading to improved self-care behaviors and independence in daily living activities among older adults^55^. By implementing continuing education initiatives, older adults can maintain their independence and enhance their self-care practices over time^56^.

The notable finding was that significant changes were observed in the control group, albeit to a lesser extent compared to the other two groups. These changes in the control group's scores were attributed to three factors: routine care, the presence of the researcher, and the testing effect. Studies have demonstrated that the presence of an observer can induce behavioral changes and impact performance and tasks, a phenomenon known as the Hawthorne effect^57^. Studies have shown that the presence of observers can influence behaviors and outcomes among older adults^58,59^. Understanding and accounting for the Hawthorne effect when working with older adults is crucial to ensure the effectiveness and accuracy of interventions and research outcomes tailored to this demographic group.

The main strengths of this study were the utilization of a three-arm, double-blind, cluster-randomized controlled trial, a combined intervention program, and low attrition rates. Education, counseling, and feedback were conducted through the social media platform "WhatsApp," enhancing accessibility for older adults. The study faced several limitations, notably due to the self-report nature of the questionnaires, which introduced the possibility of social desirability bias. Completing questionnaires through a telephone interview may yield different results compared to an in-person interview. Additionally, due to the COVID-19 outbreaks, educational interventions conducted solely online through cyberspace may lead to varied findings compared to in-person interventions or a combination of methods. While the study was double-blind, only participants and analysts were blinded, highlighting the need to blind researchers in similar randomized controlled trials.

## Conclusion

The study emphasizes the significance of integrating holistic approaches into diabetes care programs by showcasing the effectiveness of combining mindfulness and self-regulation interventions to enhance self-regulation and reduce self-neglect in older diabetic patients. Planners and policymakers can utilize the findings from the studies to advocate for the integration of cognitive and behavioral interventions, along with online training programs, to enhance self-care practices among older people with diabetes. Additionally, the study's emphasis on the long-term benefits of continuing education programs in maintaining changes in self-care behaviors can guide policymakers in developing sustainable healthcare strategies for older adults with diabetes. Insights into self-regulation and self-neglect, along with the benefits of mindfulness and self-regulation interventions, offer valuable guidance for improving self-care practices among older people with diabetes across diverse demographics and cultural backgrounds. In future research on diabetes self-management among older people, it is recommended to conduct longitudinal studies to assess the lasting effects of interventions, consider cultural influences on intervention acceptance, explore technology integration for intervention delivery, and tailor interventions to individual needs.

### Supplementary Information

Supplementary Table S1.

## Data Availability

The datasets used and analyzed during the current study are available from the corresponding author upon reasonable request.
