# Supplementary material for: Mindfulness and self-regulation intervention for improved self-neglect and self-regulation in diabetic older adults
Source: Sci Rep. 2024 Jun 15;14:13857. doi: 10.1038/s41598-024-64314-y (PMC11180124; doi:10.1038/s41598-024-64314-y)
Supplement: Supplementary file 1 — Supplementary Table S1. [file 41598_2024_64314_MOESM1_ESM.docx]

| **Table S1:** One participant’s home workout plan. | | | | | | | | |
| --- | --- | --- | --- | --- | --- | --- | --- | --- |
| **Home workout plan**  Name: Participant 23  Self-care start date: January 1, 2021  Target date: February 1, 2021 | | | | | | | | |
| Your problem | Smoking 3 times a day | | | | | | | |
| Goal setting | until the end of this month, I will only smoke half a cigarette a day | | | | | | | |
| Action planning | I try to smoke only half a cigarette a day or do some physical activity like brisk walking when I am tempted to smoke. | | | | | | | |
| Self-monitoring | Saturday | Sunday | Monday | Tuesday | Wednesday | Thursday | Friday | Feedback |
| First week | I smoked two cigarettes | I smoked two cigarettes | I smoked two cigarettes | I smoked two cigarettes | I smoked two cigarettes | I smoked two cigarettes | I smoked two cigarettes | Good job |
| Second week | I smoked one and a half | I smoked one and a half | I smoked one and a half | I smoked one and a half | I smoked one and a half | I smoked one and a half | I smoked one and a half | Good job |
| Third week | I smoked one cigarette | I smoked one cigarette | I smoked one cigarette | I smoked one cigarette | I smoked one cigarette | I smoked one cigarette | I smoked one cigarette | Good job |
| Forth week | I smoked half a cigarette | I smoked half a cigarette | I smoked half a cigarette | I smoked half a cigarette | I smoked half a cigarette | I smoked half a cigarette | I smoked half a cigarette | Perfect |
| Evaluation | You are a strong-willed person and you can completely quit smoking by the end of next month | | | | | | | |
